# Computer-Aided Brain Tumor Diagnosis: Performance Evaluation of Deep Learner CNN Using Augmented Brain MRI

**DOI:** 10.1155/2021/5513500

**Published:** 2021-06-13

**Authors:** Asma Naseer, Tahreem Yasir, Arifah Azhar, Tanzeela Shakeel, Kashif Zafar

**Affiliations:** ^1^University of Management and Technology, Lahore, Pakistan; ^2^National University of Computer and Emerging Sciences, Lahore, Pakistan

## Abstract

Brain tumor is a deadly neurological disease caused by an abnormal and uncontrollable growth of cells inside the brain or skull. The mortality ratio of patients suffering from this disease is growing gradually. Analysing Magnetic Resonance Images (MRIs) manually is inadequate for efficient and accurate brain tumor diagnosis. An early diagnosis of the disease can activate a timely treatment consequently elevating the survival ratio of the patients. Modern brain imaging methodologies have augmented the detection ratio of brain tumor. In the past few years, a lot of research has been carried out for computer-aided diagnosis of human brain tumor to achieve 100% diagnosis accuracy. The focus of this research is on early diagnosis of brain tumor via Convolution Neural Network (CNN) to enhance state-of-the-art diagnosis accuracy. The proposed CNN is trained on a benchmark dataset, BR35H, containing brain tumor MRIs. The performance and sustainability of the model is evaluated on six different datasets, i.e., BMI-I, BTI, BMI-II, BTS, BMI-III, and BD-BT. To improve the performance of the model and to make it sustainable for totally unseen data, different geometric data augmentation techniques, along with statistical standardization, are employed. The proposed CNN-based CAD system for brain tumor diagnosis performs better than other systems by achieving an average accuracy of around 98.8% and a specificity of around 0.99. It also reveals 100% correct diagnosis for two brain MRI datasets, i.e., BTS and BD-BT. The performance of the proposed system is also compared with the other existing systems, and the analysis reveals that the proposed system outperforms all of them.

## 1. Introduction

The brain is considered one of the most important organs, being responsible for memory, emotions, vision, motor skills, reactions, respiration, and a lot of other regulating functions of the human body. These functions are severely affected if some tumor starts growing inside the brain. This tumor is either the primary brain tumor which starts growing inside the brain, which is the growth of brain tissues itself, or a metastasis brain tumor which starts in some other part of the body and spreads to the brain [1]. Diagnosis of a tumor in the brain is very challenging as compared to a tumor from any other part of the human body. As the brain is filled with the blood-brain barrier (BBB), so the ordinary radioactive indicators are not able to capture the hyperactivity of tumor cells [1]. Therefore, Magnetic Resonance Imagery (MRI) and Computed Tomography (CT) scans are regarded as the best diagnostic tracers to capture disruption in BBB.

For different age groups, almost 7 to 11 persons per 100,000 get brain tumor annually [2, 3]. The Global Burden of Disease (GBD) reports almost 227,000 deaths per year due to this deadly disease. Besides, almost 7.7 million persons who survived are spending a life with disability adjustment [3]. Early detection of brain tumor not only helps in saving lives but also helps in eliminating the chance of disabilities. With early diagnosis, there will be lesser manipulation and surgical removal from the brain which is the most sensitive part of the body [4]. The manual diagnosis of the disease requires a radiologist to record a 3D image for initial insight. Then, an expert doctor is engaged for image examination and treatment planning. Unfortunately, the studies [5] investigating the accuracy of manual brain tumor diagnosis reports a disagreement between expert reviewers. The maximum agreement between the experts for manual diagnosis of brain tumor is reported between 90% and 95%. For mixed categories of tumor, mixed glioma, and medulloblastoma, the disagreement between the experts further decreases to 77% and 58%, respectively [5].

With the evolution of medical imaging technologies (MRI, CT scan, etc.) and the development in digital image processing, computer-aided diagnosis (CAD) of tissues and tumors has increased [6]. For such diagnosis systems, MRI is preferred as there is no risk of ionising radiation, and it can detect blood flow in veins accurately [7]. In the past few years, different techniques have been proposed for CAD systems for brain tumor, such as fused vectors [8], Support Vector Machine (SVM) [9, 10], transfer learning [11], and deep networks (NWs) [12]. With the recent developments in deep NWs, Convolution Neural Network (CNN) has been widely used for different CAD systems [13–16].

CNN is a sequence of multiple layers where each layer extracts features and transforms a complex input into an activation form, using partial differential functions. The layers are built on the top of each other. CNN architecture has three basic layers, i.e., a convolution layer, a pooling layer, and a fully connected layer. Where the convolution layer extracts features gradually, pooling layers downsample along the spatial domain, and the fully connected layer classifies. A vanishing gradient problem may rise when small numbers appear while computing gradients. To avoid vanishing gradient difficulty, a Rectified Linear Unit (ReLU) layer is also added after each convolution layer, as an element-wise activation function. Some other CNN layers are the input layer, the dropout layer, the output layer, and the network in network layer [17, 18].

In this article, a computer-aided brain tumor diagnosis tool is proposed to examine brain MRIs and provide an early diagnosis with improved performance. For this proposed CAD system, CNN is trained on the BR35H::Brain Tumor Detection 2020 dataset [19], and its performance is evaluated for six different brain tumor MRI datasets [20–25]. The trained CNN model achieves 100% accuracy for two datasets, i.e., Brain Tumor Segmentation (BTS) [24] and BD-BrainTumor (BD-BT) [23]. For the performance evaluation of the model, different statistical evaluation methods are used including sensitivity, specificity, precision, recall, *f*_measure_, false positive (FP) ratio and Receiver Operating Characteristic (ROC) curve.

The major contributions of this research are as follows:

- An outperforming CNN-based computer-aided brain tumor diagnosis system for an early and reliable detection of brain tumor to assist in the rapid treatment planning
- A technique consisting of preprocessing on images, feature extraction, reduction of feature space, and finally classification of images into positive and negative diagnoses of brain tumor
- Improvement in CNN performance by applying geometrical and statistical data augmentation techniques on brain tumor MRI
- Evaluation of the power of CNN by classifying totally unseen data, consisting of the six latest brain tumor MRI datasets
- Producing up to 100% accuracy by the trained CNN model
- Achieving a high average sensitivity, i.e., 0.99 for all the datasets
- A comprehensive comparative analysis with the other existing systems

The paper is arranged in such a way that Section 2 presents related work, Section 3 describes proposed methodology, Section 4 portrays results and discussion, and Section 5 concludes the research along with the future work.

## 2. Related Work

CNN has been widely used for solving different problems in different areas [17, 18] but, for the processing of images for health applications, its performance is remarkable. A lot of research exists in which CAD-based diagnosis of diseases is proposed. For the detection of brain tumor, a CNN with a neutrosophic is explored [26]. In this hybrid technique, features are extracted by CNN, and for classification, Support Vector Machine (SVM) and K-Nearest Neighbors (KNN) are used. The system is trained and tested on only 160, half negative and half positive, images. Using fivefold cross-validation, 95.62% accuracy is achieved by the proposed technique.

In another research, brain tumor is detected by using both handcrafted features and the features extracted by the deep learner [8]. In the proposed system, a transfer learning model acquires features while shape and texture are extracted manually. For classification, entropy and fused vectors are fed to the classifier. In another research, brain tumor is classified by using CNN and transfer learning [13]. For this experiment, pretrained GoogLeNet is used to extract features. For classification, already proven classifiers are used. By using fivefold cross-validation, 98% accuracy is achieved. CNN is trained on augmentation of extensive data for brain tumor classification [15]. In the proposed system, tumor area is segmented by using a deep learning technique. The research uses a pretrained CNN model and evaluates the performance of the system on original as well as augmented data.

In the proposed system, brain tumor MRIs are used to train CNN [16]. In this research, CNN architecture is designed by using a hypercolumn technique. An attention module identifies the area of interest before transferring it to CNN layers. The proposed system achieves 96.05% accuracy. CNN is also used for the segmentation of brain tumor in MRI [14]. The results of a clustering algorithm, traditional classifiers, and CNN are compared. Traditional classifiers include Multilayer Perceptron (MLP), Support Vector Machine (SVM), Logistic Regression, K-Nearest Neighbor (KNN), Random Forest, and Naïve Bayes. The performance of CNN, with 97.87% accuracy, is reported as the best among all the classifiers. For combining texture and structural information in the four MRI sequences, a fusion process is implemented for brain tumor detection [27]. A fusion process uses Discrete Wavelet Transformation (DWT). By using the Daubechies wavelet, more information from the tumor region is extracted. After preprocessing, CNN is used for classification of tumor and nontumor regions. As per results, fused images reveal better performance. In another research, six CNN models are trained for brain tumor detection [9]. The architecture of the CNN models is defined on the basis of different hyperparameters. The results show a better performance by deep learning models as compared to the conventional methods. In another similar approach, different architectures for CNN models are designed for classifying benign tumor [10]. The accuracy for different models is reportedly between 96% and 99%.

In a study, normal brain tissues are differentiated from brain tumor and pseudobrain tumor by using LSTM [28]. Different augmentation techniques are applied on an MRI signal dataset for training stacked Bi-LSTM. Using 5-fold cross-validation, average accuracy achieved by the proposed technique is 91.46%. A multiscale Deep CNN [29] is proposed which can analyse tumor MRIs and classify them into glioma, meningioma, and pituitary tumor. The performance of the proposed model is evaluated on an MRI image dataset consisting of 3,064 images. Classification accuracy of the proposed CNN is reported as 97.3%. Deep network ResNet-50 is trained on 3,064 brain MR images taken from three brain MRI datasets [30]. The performance of the model is evaluated with the help of a key performance matrix. The proposed model achieves 97.08% average accuracy for nonaugmented data and 97.48% average accuracy for augmented data. In another study, eight CNN models [31] are developed and trained on brain MRI for a CAD system of brain tumor. CNN models reveal accuracy between 90% and 99%. A 3D CNN model is proposed to extract features from brain MRIs [32]. The features extracted by CNN are provided to a correlation-based model for optimum feature selection, and a feed-forward ANN is used for classification. The accuracy achieved by the proposed technique is 92.67%, 96.97%, and 98.32%, for three different datasets.

## 3. Proposed Methodology

The focus of the current research is on computer-aided diagnosis of brain tumor by feeding brain tumor MRIs to CNN. Using labelled data, CNN extracts features and learns to classify images as positive or negative diagnosis of brain tumor. This supervised model of CNN uses preprocessed images for a better performance. The main phases of research include, gathering the latest brain tumor image dataset, preprocessing on images, gradual and incremental training of the model, and finally performance evaluation by testing the model on six different unseen MRI datasets.

### 3.1. Datasets

**Figure 1:**
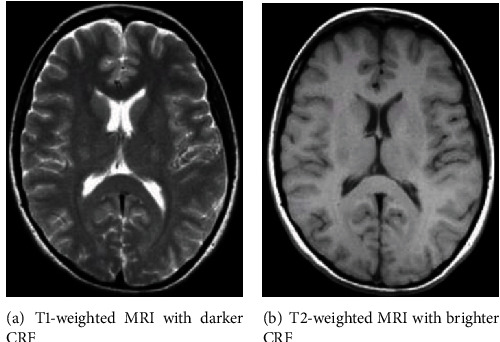
Samples of T1-weighted and T2-weighted brain MRI with different intensity values of cerebrospinal fluid (CRF).

In the brain MRI images, brain tissues can be represented by either T1 or T2 relaxation time. T1-weighted images use short Repetition Time (TR) and Time to Echo (TE) while T2-weighted images use longer TR and TE times. The time taken in milliseconds by T1- and T2-weighted scanning is given in Table 1. The training data that is used in this research contains both T1-weighted and T2-weighted images, while the datasets used for testing contain either T1-weighted images or T2-weighted images. The sample T1-weighted and T2-weighted images are described in Figures 1(a) and 1(b), respectively.

The signals generated by loop coils of the MRI device are digitized by Fast Fourier Transformation (FFT) which provides real value, imaginary value, magnitude, and phase. All the datasets, used in this research, are based on the magnitude of FFT.

The performance of machine learning and deep learning techniques highly depends on the dataset and its size. The uniqueness of this work is to train the model on a small dataset in such a way that it can be sustainable for unseen data which is the exact target of these learning systems. Therefore, in this research, almost only 28% data is used for training the model while the rest of the data is used for testing to assure the robustness of the proposed CAD system.

#### 3.1.1. Training Dataset

For training CNN, the BR35H::Brain Tumor Detection 2020 (BR35H) [19] dataset is used which contains 255 negative and 255 positive MRIs of brain tumor. 90% of the images from this dataset are used for training the model. The dataset contains both T1-weighted and T2-weighted image sequences. The usability rating of this dataset is reported as 7.5 [19]. Data usability rating is calculated on the basis of licensing, tagging, overview of data, and its description, ease, maintainability assurance, machine readable file formats, metadata, and availability of public kernel.

#### 3.1.2. Testing Datasets

For the performance evaluation of CNN, six totally unseen datasets are used including Brain MRI Images for Brain Tumor Detection (BMI-I) [21], Brain Tumor Image Dataset (BTI) [25], Brain MRI Images for Brain Tumor Detection (BMI-II) [22], Brain Tumor Segmentation (BTS) [24], Brain MRI Images for Brain Tumor Detection (BMI-III) [20], and BD-BrainTumor (BD-BT) [23]. An overview of these datasets is given in Table 2.

*(1) BMI-I*. The BMI-I dataset contains, in total, 171 images out of which 86 images are positive for brain tumor and 85 images are negative. The images in this dataset are T1-weighted, and the usability of this dataset is reported as 5.0.

*(2) BTI*. The BTI dataset consists of 20 images with 50% positive and 50% negative class labels. The images in this dataset are T2-weighted, and the usability of this dataset is reported as 4.4.

*(3) BMI-II*. There are 92 images which are taken from the BMI-II dataset. 86 images are labelled as positive and 6 images are labelled as negative. The images in this dataset are T1-weighted, while the usability ranking of this dataset is 3.8.

*(4) BTS*. 140 images from the BTS dataset, used in this research, consists of the same number of positive and negative images. The images in this dataset are T2-weighted, and the usability of this dataset is reported as 3.1.

*(5) BMI-III*. BMI-III contains 86 positive and 85 negative images. The images are T1-weighted, and the usability of this dataset is 1.3.

*(6) BD-BT*. 671 images, all positive, are used from BD-BT dataset. The images in this dataset are T2-weighted, while the usability ranking of this dataset is reported as 2.5.

### 3.2. Data Augmentation and Preprocessing

**Figure 2:**
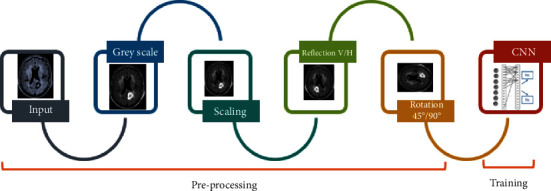
Preprocessing steps: greyscale conversion, scaling, rotation, and reflection.

**Figure 3:**
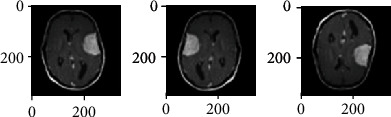
Original image, image with horizontal reflection, and image with vertical reflection.

In this research, a total of 1,775 images were used for training and testing. All the images are preprocessed before feeding them to CNN, as described in Figure 2. At first, these images are converted into single-channel images, known as greyscale images. After colour data augmentation, geometric data augmentation including scaling, flipping, and rotation is applied. As the original images are in different sizes, so they are rescaled to a size of 256 × 256. To make CNN perform dynamically for different datasets, image reflections, in both horizontal and vertical dimensions, are generated by using equation (1) and equation (2). The effects of such reflections can be seen in Figure 3.

(1) $$\begin{array}{c} \text{B}{\text{T}}_{\text{h}}\left(x,y\right)=\text{BT}\left(-x,y\right), \end{array}$$

(2) $$\begin{array}{c} \text{B}{\text{T}}_{\text{v}}\left(x,y\right)=\text{BT}\left(x,-y\right), \end{array}$$

where BT is the original MRI of the brain, BT_h_ is the horizontal reflection, and BT_v_ is the vertical reflection.

**Figure 4:**
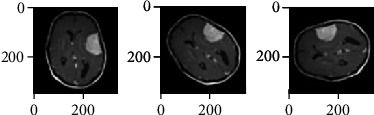
Original image, image with 45° degree rotation, and image with 90° degree rotation.

After getting the reflected images, two more types of images are generated by rotating the original images at 45° and 90°. For rotations, equation (3) and equation (4) are used. The effects of these rotations are illustrated in Figure 4.

(3) $$\begin{array}{c} R_{\theta }=\left[\begin{array}{cc} \cos\theta & -\sin\theta \\ \sin\theta & \cos\theta \end{array}\right], \end{array}$$

(4) $$\begin{array}{c} {\text{BT}}_{\text{r}}={\text{BTR}}_{\theta }, \end{array}$$

where BT is the original MRI of the brain, BT_r_ is the rotated image, *R*_*θ*_ is the rotation matrix, and *θ* is set as 45° and 90°.

As the brain tumor MRI datasets used in this research are from seven different sources and are in different formats, so further preprocessing is applied before feeding these images to CNN. To harmonize these images, standardization is performed by using equation (5), equation (6), and equation (7). With the help of the standardization process, all the images get 0 as mean *μ* and 1 as standard deviation *σ*. Turning *μ* into zero makes different datasets comparable. On the other hand, *σ* with a value of 1, makes the data distribution comparable to a normal distribution.

(5) $$\begin{array}{c} \mu =\frac{\sum_{x=1}^{M}\;\sum_{y=1}^{N}\;{\text{BT}}_{x,y}}{M\times N}, \end{array}$$

(6) $$\begin{array}{c} \sigma =\sqrt{\frac{1}{M\times N}\overset{M}{\underset{x=1}{\sum }}\;\overset{N}{\underset{y=1}{\sum }}\;{\left({\text{BT}}_{x,y}-\mu \right)}^{2}}, \end{array}$$

(7) $$\begin{array}{c} {\text{BT}}_{\text{s}}=\text{BT}-\frac{\mu }{\sigma }, \end{array}$$

where BT is the brain tumor image, *M* and *N* are its dimensions, BT_s_ is the standardized brain tumor image, *μ* is mean, and *σ* is standard deviation.

Finally, all the images with negative labels are mapped to the value of 0, and the ones with a positive label are mapped to the value of 1, for the supervised training of CNN.

### 3.3. Convolution Neural Network (CNN) Architecture

**Figure 5:**
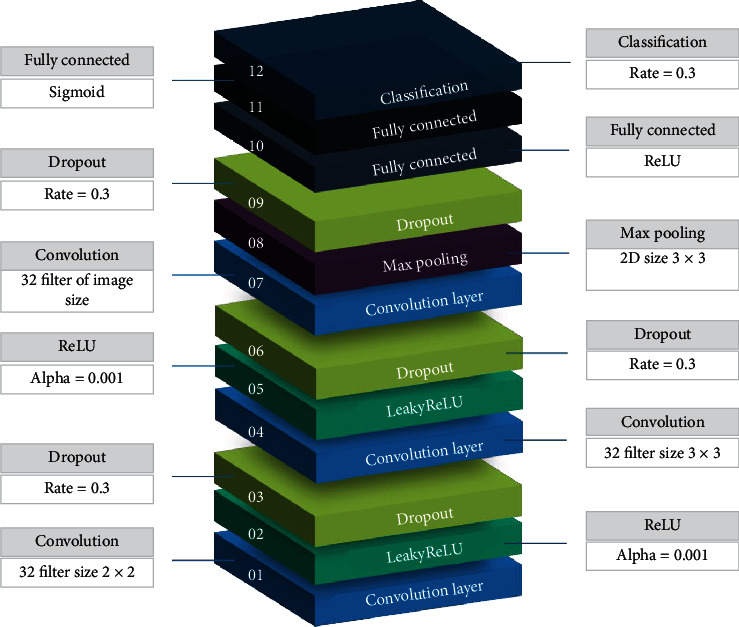
Layers of the proposed convolution neural network architecture.

The architecture of CNN is defined sequentially, and the model is built layer by layer. The first layer is the input layer, consisting of a size defined by the input images. The next layer is the 2D convolution layer with 32 filters and a 2 × 2 kernel. A Rectified Linear Unit (ReLU) computes cheaply and converges quickly. To avoid the “dyingReLU” problem, i.e., zero ReLU for negative values, LeakyReLU, with alpha set as 0.001, is added on the top of the convolution layer. After this layer, the dropout layer is added with a dropout rate set as 0.3. Once this architecture is defined, a 2D convolution layer is again created using a kernel function with a size of 3 × 3 followed by LeakyReLU and dropout layers with the previously defined parameters. After these layers, another 2D convolution layer is added with the number of filters set as per the image size. After that, a pooling layer is added to reduce and summarise the feature map by downsampling. To prevent the CNN model from overfitting, a dropout layer is added to the network after the pooling layer. After this layer, a flattening technique is defined for the output of the network followed by two fully connected dense layers with units 12 and 1. LeakyReLu and sigmoid are used as activation functions in these fully connected layers, respectively. The architecture of CNN implemented in this research is illustrated in Figure 5.

Convolution operations are performed by applying equation (8), and for nonlinearity, ReLU layers are implemented with equation (9).

(8) $$\begin{array}{c} X\left[i,j\right]=\left(w\times x\right)\left[i,j\right]=\overset{M}{\underset{s=-M}{\sum }}\;\overset{N}{\underset{t=-N}{\sum }}\;x\left[i+s,j+t\right]w\left[s,t\right], \end{array}$$

(9) $$\begin{array}{c} f\left(x\right)=\left\{\begin{array}{cc} x, & \text{if }x\geq 0,\\ \alpha x, & \text{otherwise}, \end{array}\right. \end{array}$$

where *x* is the input image of brain tumor, *w* is the kernel or convolution operator, *X* is the feature map of processed data, kernel size is *M* × *N*, and *i* and *j* are the row and the column at *i*th and *j*th position of input *x*. The value of *α* is set as 0.001.

The dropout layer regularises the deep learners to avoid overfitting. This layer can be applied on a fully connected layer or a convolutional layer. The effect on a fully connected layer would be in the shape of dropping out neurons to avoid overfitting, while the effect on the convolutional layer would be in the shape of adding noise into the feature maps. In the preprocessing phase, normalisation and data augmentation are already applied on the training data, hence, the dropout layer is not applied on fully connected layers, rather it is applied on the convolution layer to generate effects on feature maps. In this way, the effect of the augmented data on overfitting is evaluated without dropping out neurons.

#### 3.3.1. Padding and Stride

Due to convolution operations at convolutional layers, some pixels from the boundary of the images are lost, thus resulting in different input and output image sizes. The loss of pixels depends on kernel size. If the size of kernel is (*n* × *m*) and *n* is even, then (*n*/2) rows from the top and bottom of the image will be lost. In case *n* is odd, then the loss will be of (*n*/2) − 1 rows from the top and bottom. The same is true for the first and last *m* number of columns. To overcome this loss, an elementary solution is to add extra pixels all over the boundary. In the proposed CNN, zero padding is used, considering the size of the kernel. In the case of even values of *m* and *n*, (*n*/2) × (*m*/2) zero padding is applied, and for odd values of *m* and *n*, ((*n*/2) − 1) × ((*m*/2) − 1) zero padding is applied around the image boundary. For convolving with the image, the kernel window slides on the whole image step by step. Stride is the component of CNN which decides the size of step. In the proposed CNN, the value of stride is set as 1, for both rows and columns.

### 3.4. Training

After preprocessing the images and designing all the layers of the CNN architecture, the deep network CNN is trained on rescaled and preprocessed original images along with their reflected and rotated set of images from dataset BR35H. For training the model, sigmoid activation function, given in equation (10), is used as an optimiser due to its nature of smoother output while observing smaller changes in the input. The learning rate of the model is set as 0.001.

(10) $$\begin{array}{c} f\left(x\right)=\sigma \left(x\right)=\frac{1}{1+e^{-x}}. \end{array}$$

For training CNN for binary classification of brain tumor MRIs, cross entropy, given in equation (11), is used as a loss function.

(11) $$\begin{array}{c} \text{CE}=-\overset{c^{\prime }=m}{\underset{i=1}{\sum }}\;l_{i}\log\left(s_{i}\right)=l_{1}\log\left(s_{1}\right)-\left(1-l_{1}\right)\log\left(1-s_{1}\right), \end{array}$$

where *l*_*i*_ and *s*_*i*_ are the CNN scores for each positive and negative class, while the value of *m* is 2 (binary classifier).

Instead of training CNN in one go, a step by step gradual training is performed in six phases, and its performance on the validation set is evaluated before entering in the testing phase. For validation, 10% of data from the BR35H dataset is used after shuffling the images. At first, CNN is trained only for 10 epochs. After 10 epochs, 79.36% binary classification accuracy is achieved on the validation set. Due to such low training accuracy, CNN is again trained for 5 more epochs. To enhance the training accuracy of the deep network, it is gradually trained for three more times with 10, 20, and again 20 epochs. The increase in training accuracy with more training of the model is described in detail in Table 3. After the 49th epoch, CNN shows 100% training accuracy on the validation set.

**Figure 6:**
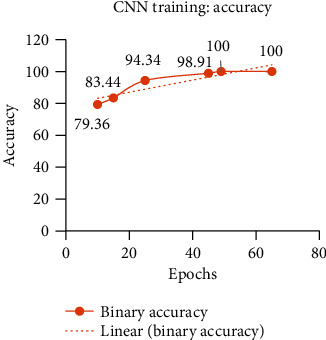
Training accuracy curve of the CNN model showing the gradual progress of the deep neural network.

**Figure 7:**
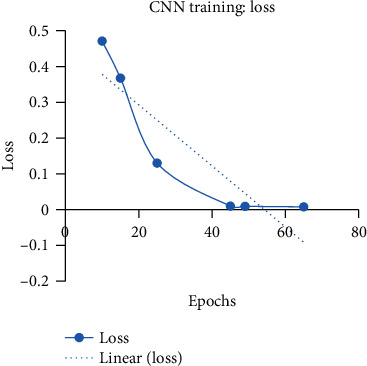
Loss curve during the training of the CNN model showing a good learning rate.

At each time, the training of the CNN model is made with a fixed number of epochs. The two curves of training accuracy and training loss are monitored. If the curves show an overall monotonic increase in accuracy and monotonic decrease in loss, then the model is further trained for another fixed number of epochs. The training of the CNN model is stopped when 100% training accuracy is achieved. The gradual increase in accuracy and decrease in loss during the 65 epochs of training are illustrated in Figures 6 and 7, respectively.

## 4. Results and Discussion

For this research, only one MRI dataset of brain tumor is used for training CNN, while six datasets are used for testing the performance of the model. In this way, almost only 28% of the data is used for training and validation, while 72% of the data is used for testing. The performance of CNN is evaluated by precision, recall/sensitivity, *f*_measure_, and specificity as given in equation (12), equation (13), equation (14), and equation (15).

(12) $$\begin{array}{c} \text{Precision}=\frac{\text{TP}}{\left(\text{TP}+\text{FP}\right)}, \end{array}$$

(13) $$\begin{array}{c} \text{Recall}=\text{sensitivity}=\frac{\text{TP}}{\left(\text{TP}+\text{FN}\right)}, \end{array}$$

(14) $$\begin{array}{c} f_{\text{measure}}=\frac{2\times \text{precision}\times \text{recall}}{\text{precision}+\text{recall}}, \end{array}$$

(15) $$\begin{array}{c} \text{Specificity}=\frac{\text{TN}}{\left(\text{TN}+\text{FP}\right)}. \end{array}$$

**Figure 8:**
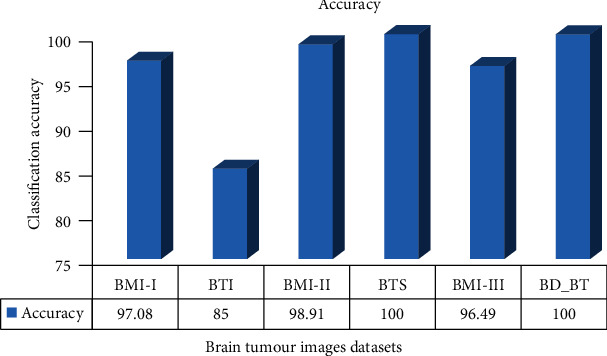
Accuracy of the proposed Convolution Neural Network for all the six test datasets.

The accuracy of CNN for all the datasets is above 96%, except for the dataset BTI, as described in Table 4. For the BTS and BD-BT datasets, the model has classified the brain tumor images with 100% accuracy. The performance of the model is consistent for the six datasets, excluding BTI, which contains only 20 images out of the 1265 tested images. The accuracy of each test dataset is illustrated in Figure 8.

**Figure 9:**
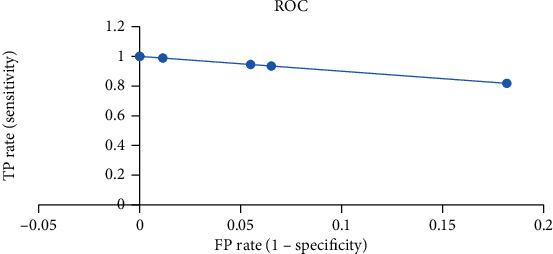
Receiver Operating Characteristic (ROC) curve between True Positive Rate (TPR) and False Positive Rate (FPR).

The research also reveals that the performance of CNN is very remarkable for positive class images, as out of 1009 positive class images only 14 images are missclassified as negative. CNN performance for positive images is also visible in the test results of the BD-BT dataset which contains 671, all positive images, classified correctly. Even for negative class images, the performance of CNN is quite reliable as only 1 image, out of 242 negative images, is classified as false positive. The average accuracy of CNN, for all the six datasets, is 98.8%. For analysing TP rate vs. FP rate, ROC is also plotted in Figure 9.

The architecture of CNN, defined in this research to diagnose brain tumor with the help of preprocessed brain MRI, is able to achieve reliable accuracy. As compared to the other latest research work [10, 13, 14, 16, 26], where CNN models are trained on 80% of data and accuracy is still less than 100%, the CNN model, designed and trained in this research, reveals a better performance. The model is able to achieve 100% accuracy on two datasets [23, 24]. Even the accuracy reported by using pretrained CNN models [13, 15] is not better than the CNN model that is trained in this research.

The outstanding performance of CNN is due to different factors. The architecture of CNN, designed in this research, contains three convolution layers. Convolution layers, the basic building blocks of the network, merge different sets by convolving images with the convolution filter, thus creating a feature map. In the proposed architecture, three layers are designed for extracting feature maps for producing more information for classification. The decision regarding hyperparameters, such as filter size and filter count, also plays a vital role in the learning phase of the network. For better learning, CNN is trained in a such a way that overfitting can be avoided for which an augmented data technique is used. For augmented data, different transformations such as rotation and reflection are applied to the input images. Due to data augmentation via transformations, regularisation of data is achieved which ultimately leads CNN to learn in a generic way instead of remembering only training data. Due to this factor, CNN avoids overfitting and performs better even for unseen datasets from different resources. Another factor which helped to achieve better accuracy is data cleaning, for which mean normalisation is applied to the input images. It enables CNN to compare different datasets and perform remarkably. Besides, dropout layers, which regularise CNN to avoid overfitting, are also applied on convolution layers which add noise to feature maps. Adding such an effect into feature maps makes CNN more robust and sustainable.

### 4.1. Comparative Analysis with the Other Systems

**Figure 10:**
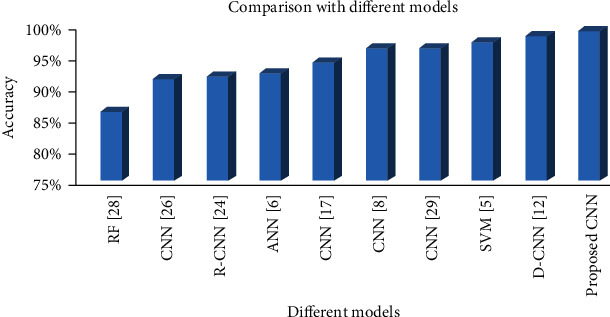
Comparison between the performance of the proposed CNN model and the other existing models.

The performance of the proposed system is compared with the other most recent computer-aided brain tumor diagnosis systems. In these systems, CNN [33–37], Random Forest [38], Artificial Neural Network (ANN) [39], Deep CNN (D-CNN) [40], Support Vector Machine (SVM) [41], and Faster Region-based CNN (R-CNN) [35] are used. Table 4 gives an overview of the performance revealed by these systems. Least accuracy at 86% is shown by the Random Forest Classifier. Except for D-CNN, the accuracy achieved by CNN-based systems is between 91% and 96%. Only D-CNN has achieved 98.07% accuracy which is still below the accuracy that is revealed by the system proposed in this research. A clear comparison of the all these systems is portrayed in Table 5. Among these nine systems, six are CNN-based but still none of them is performing better than the system designed and proposed in this research. The CNN model with deep layers and data augmented MRIs has outperformed all the other systems as illustrated in Figure 10.

## 5. Conclusion and Future Work

In this research, a CNN based computer-aided diagnosis system of brain tumor is proposed. The deep network model CNN is trained only on 28% of data, and its performance is analysed on 72% of totally unseen data which is taken from different brain tumor MRI datasets. The model has provided, on average, 98.81% correct diagnosis of brain tumor while achieving 100% accuracy for two datasets. In the future, the performance of this CNN-based CAD system can be further enhanced by conducting further research and exploring other deep networks, variations of CNN, feature maps, and augmentation techniques.

## Figures and Tables

**Table 1:** Time in milliseconds, taken by T1-weighted image scanning and T2-weighted image scanning.

	T1-weighted (short TE and TR)	T2-weighted (long TE and TR)
TE	14 ms	90 ms
TR	500 ms	4000 ms

**Table 2:** Brain MRI datasets.

		Brain tumor images datasets	Positive	Negative	Total
Training	BR35H	BR35H::Brain Tumor Detection 2020	255	255	510

Testing	BMI-I	Brain MRI Images for Brain Tumor Detection	86	85	171
	BTI	Brain Tumor Image Dataset	10	10	20
	BMI-II	Brain MRI Images for Brain Tumor Detection	86	6	92
	BTS	Brain Tumor Segmentation	70	70	140
	BMI-III	Brain MRI Images for Brain Tumor Detection	86	85	171
	BD-BT	BD-BrainTumor	671	0	671
		Total	1264	511	1775

**Table 3:** Loss and accuracy trends during the gradual training of CNN model.

No. of epochs/phase	Total epochs	Loss	Accuracy
10	10	0.4711	79.36
5	15	0.3675	83.44
10	25	0.13	94.34
20	45	0.0098	98.91
20	49	0.0091	100
	65	0.0077	100

**Table 4:** Precision, recall, and *F*_measure_ with *α* = 0.5, for six testing datasets.

Datasets	Total	TP	TN	FP	FN	Precision	Recall	*F*_measure_ *α* = 0.5
BMI-I	171	86	80	0	5	1	0.946	0.973
BTI	20	9	**8**	1	2	0.9	0.819	0.86
BMI-II	92	86	**5**	0	1	1	0.989	0.995
BTS	140	70	**70**	0	0	1	1	1
BMI-III	171	86	**79**	0	6	1	0.935	0.968
BD-BT	671	671	**0**	0	0	1	1	1
Total	**1265**	**1008**		**1**	**14**	**—**	**—**	**—**

**Table 5:** Comparative analysis of the proposed system with the other CAD systems.

Reference	Technique	Training images	Testing images	Accuracy
[38]	Random Forest Classifier	372	93	86%
[36]	CNN	2451	613	91.30%
[35]	R-CNN	2451	613	91.66%
[39]	ANN	160	40	92.14%
[34]	CNN	222	56	93.9%
[33]	CNN	400	100	96.08%
[37]	CNN	2451	613	96.13%
[41]	Support Vector Machine (SVM)	372	93	97.1%
[40]	Deep CNN (D-CNN)	372	93	98.07%
Proposed model	CNN	**510**	**1265**	**98.8%**

## Data Availability

The datasets analysed during the current research are available at the links given below: (1) BR35H::Brain Tumor Detection 2020: https://www.kaggle.com/ahmedhamada0/brain-tumor-detection. (2) Brain MRI Images for Brain Tumor Detection: https://www.kaggle.com/navoneel/brain-mri-images-for-brain-tumor-detection. (3) Brain Tumor Images Dataset: https://www.kaggle.com/simeondee/brain-tumor-images-dataset. (4) Brain MRI Images for Brain Tumor Detection: https://www.kaggle.com/jjprotube/brain-mri-images-for-brain-tumor-detection. (5) Brain Tumor Segmentation: https://www.kaggle.com/leaderandpiller/brain-tumor-segmentation. (6) test-brain: https://www.kaggle.com/monagaffer12345/test-brain. (7) BD-BrainTumor: https://www.kaggle.com/dorianea/bd-braintumor
